# Developing GLAD Parameters to Control the Deposition of Nanostructured Thin Film

**DOI:** 10.3390/s22020651

**Published:** 2022-01-14

**Authors:** Jakub Bronicki, Dominik Grochala, Artur Rydosz

**Affiliations:** 1 Biomarkers Analysis LAB, Institute of Electronics, AGH University of Science and Technology, al. A. Mickiewicza 30, 30-059 Krakow, Poland; jakubx@student.agh.edu.pl (J.B.); or artur.rydosz@adediabetics.pl (A.R.); 2 Advanced Diagnostic Equipment Sp. z o.o., ul. W. Weissa 7/C1, 31-339 Krakow, Poland

**Keywords:** glancing angle deposition, magnetron sputtering, thin films, metal oxides, gas sensors

## Abstract

In this paper, we describe the device developed to control the deposition parameters to manage the glancing angle deposition (GLAD) process of metal-oxide thin films for gas-sensing applications. The GLAD technique is based on a set of parameters such as the tilt, rotation, and substrate temperature. All parameters are crucial to control the deposition of nanostructured thin films. Therefore, the developed GLAD controller enables the control of all parameters by the scientist during the deposition. Additionally, commercially available vacuum components were used, including a three-axis manipulator. High-precision readings were tested, where the relative errors calculated using the parameters provided by the manufacturer were 1.5% and 1.9% for left and right directions, respectively. However, thanks to the formula developed by our team, the values were decreased to 0.8% and 0.69%, respectively.

## 1. Introduction

The next industrial revolution would not be possible without developments in nanotechnology, including both the design and fabrication process of thin films. Of the various techniques, glancing angle deposition (GLAD) offers the possibility to fabricate nanostructured columnar thin films such as metallic films, metal oxides, and metal nitrides and, therefore, GLAD can be applied in every fabrication process where the nanocolumnar shape is needed. In addition, GLAD does not require any demanding conditions in the vacuum system, and it could be applied to physical vapor deposition processes such as sputtering and evaporation. The minimal set of parameters, such as the tilt, rotation, and substrate temperature, offers high precision in the fabrication of nanoscale thin films for various applications.

Our work focuses on the fabrication of metal oxides such as TiO_2_ [1], WO_3_ [2], and CuO [3] using GLAD. These are used in manufacturing gas-sensing materials for the detection of several gases such as ammonia [4], acetone [4], and ozone [5]; they are also used in humidity sensors [6,7,8,9]. This is part of an attempt to improve the safety and quality of life of people. A good example is monitoring for diabetics, whose blood glucose measurements can be replaced by a sensor that detects diabetes biomarkers in exhaled air. Another application is the attempt to provide the public with a sense of security by installing fire-detection sensors [10]. Another example of compounds used in GLAD are metal sulphides, such as CdS, for which the properties of the obtained film are used in photovoltaics, due to them having a wide band gap, and in optical sensors as a photoconductive material [11].

Gas sensors are defined by important features, such as the selectivity and sensitivity [12,13,14]. Since selectivity is mostly related to the chemical structure of the gas-sensing materials and its capability to selectively detect target gas molecules, sensitivity relies on the physical structure described as the surface–volume ratio [15]. Therefore, the possibility to deposit a gas-sensing layer of a nanocolumnar shape under controlled conditions is a powerful tool. Moreover, physical vapor deposition can be easily adapted to complementary metal-oxide semiconductor (CMOS) technology; therefore, front-end electronics compounds can be realized by using the same technology [16,17,18]. Stability is also an important feature of gas sensors; however, more aspects than the gas-sensing layer itself are responsible for a suitable stability, e.g., gas-sensor substrate, electrodes, front-end electronic circuits.

**Figure 1:**
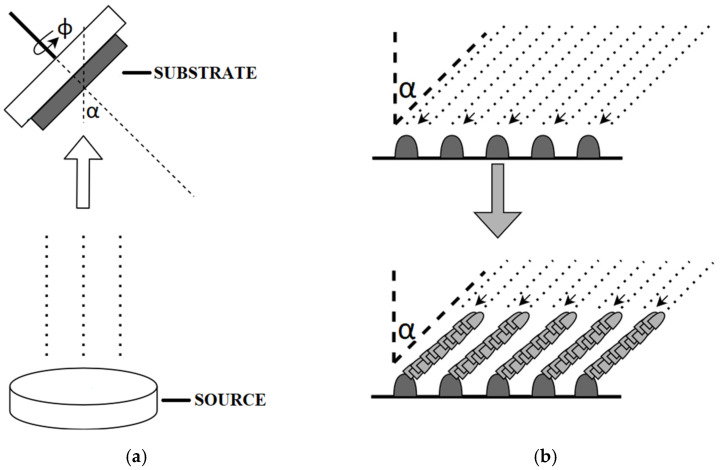
Scheme of the GLAD technique: (**a**) drawing of main process; (**b**) film deposition process on the substrate with shadowing effect.

As mentioned above, GLAD is controlled by a set of three parameters—the tilt, rotation, and substrate temperature. The general concept of the GLAD technique is shown in Figure 1a, where the manipulator rotates with substrates with velocity φ at angle α and the source of thin films, for example, magnetron targets.

The principle of this method is based on the deposition of the film at various angles, assuming that the path of the vapour with respect to the substrate is not parallel. The dimensions of the resulting column, as well as the thickness of the material, are controlled by varying the rate of rotation and the angle of inclination of the substrate. The basic principles of the various deposition methods can be found elsewhere, for example, in the works of Diederik et al. [19], Schultz et al. [20], and Mintonette et al. [21], where magnetron sputtering, e-beam, and thermal evaporation are presented, respectively. Regardless of the deposition method, the GLAD technique is characterised by several steps. At the beginning, the particles disperse randomly on the substrate, resulting in a rough surface. The growth of the columns is then ordered by ballistic shadowing effects and adatom diffusion (Figure 1b). In the final stage of the process, the columns increase in thickness, resulting in a very high solid filling factor [4,12].

**Figure 2:**
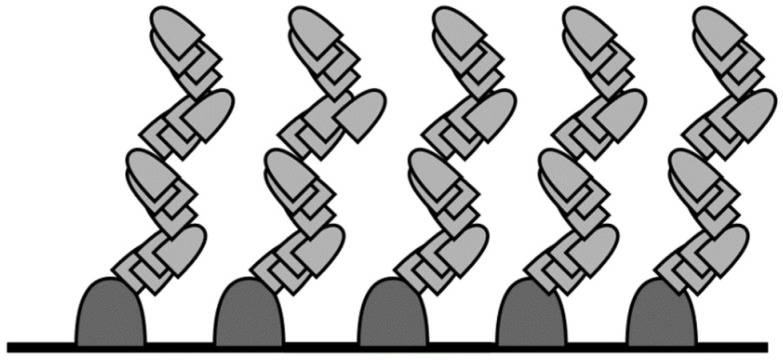
Cross section of plate with zigzag pattern.

Depending on the designed deposition process, the fabrication may require a slight change, for example, a small tilt correction or radical tilt change (e.g., for zig-zag structures as presented in Figure 2 [22,23,24]); therefore, the system should enable such a modification. Lee et al. [10] developed a deposition system based on the GLAD technique combined with e-beam evaporation for highly sensitive fire detectors. Butt et al. [25] fabricated fibre-to-chip coupling with slanted binary grating by applying certain modifications to the PhiSweep GLAD method. The authors tested the system for the deposition of a variety of columnar nanostructures, including nanorods, nanozigzags, and nanohelixes. Additionally, the number of sensors on a 100 mm wafer was 732, which proves the possibility for the mass production of such detectors. However, there is a lack of information about the process control system, which reduces the possibility of other researchers implementing a similar system. Similarly, Sakkas et al. [23] presented the tuning of the optical properties of WO_3_ films deposited by custom-made magnetron sputtering technology coupled with the GLAD technique. The authors deposited zigzag columnar microstructures that exhibited various transmissions and refractive indexes in the 400–1000 nm wavelengths. The GLAD technique was also used to deposit ZnO and low-doped ZnO:Ag films that exhibited improved room-temperature ferromagnetism effects. Such experiments were conducted by Correa et al. [26] and Santos et al. [24]. However, the authors did not mention the controlling system for the GLAD system. Recently, Limwichean et al. [27] presented a very interesting approach for the GLAD technology, where regardless of the above-mentioned parameters such as the tilt, rotation, and temperature, the additional parameter of the substrate position relative to the magnetron source was investigated in the 10–50 mm range. The authors compared the deposition rates, roughness, work functions, transmittance, and current density of the WO_3_. The obtained results showed that the substrate position should also be included for the set of the GLAD parameters. Additionally, Mendoza-Rincon et al. [28] recently presented how the substrate rotation affects the roughness in Zr thin films grown using the GLAD technique. However, in the vast majority of papers, homemade or custom-made GLAD systems are presented [29,30,31,32,33,34,35,36,37]. In these cases, the possibility of being widely used in industrial processes is lowered. Therefore, we designed the GLAD process based on the commercially available components from Kurt J. Lesker Company (described in detail in the Materials and Methods Section) with dedicated software designed by our team.

In this paper, the GLAD controller is described, including both hardware and software components for managing the GLAD deposition of metal oxide thin films for gas-sensing applications.

## 2. Materials and Methods

**Figure 3:**
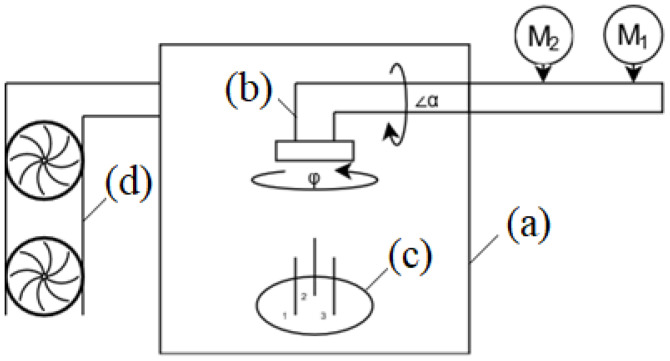
Scheme of GLAD machine, details are given in Table 1.

Within this study, a commercially available physical vapour deposition (PVD) system was used, based on components from the Kurt J. Lesker Company (Jefferson Hills, PA, USA). The crucial components are illustrated in Figure 3, including an ultra-high vacuum chamber, magnetron sources, GLAD manipulator, and pumping system.

### 2.1. Commercially Supplied: Deposition Chamber

**Figure 4:**
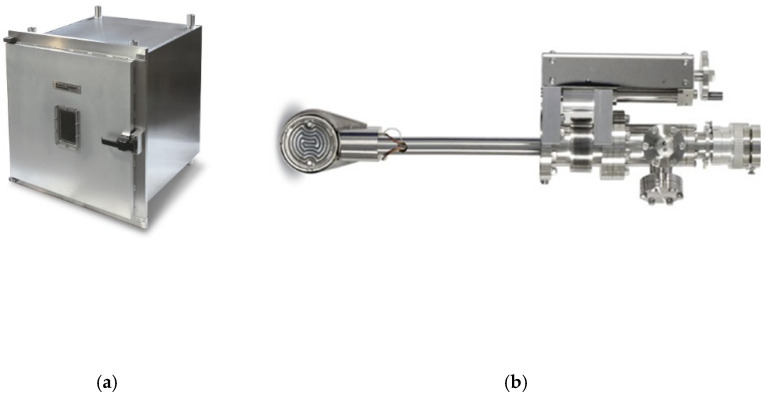
Photos of modules from the Kurt J. Lesker Company. (**a**) The ultra-high vacuum chamber [38]; (**b**) the EC-R Series manipulator [39].

A photograph of the UHV chamber is provided in Figure 4a. The chamber (640 mm × 640 mm × 780 mm) was manufactured from 304 stainless steel and was equipped with an O-ring sealed door made from 6061-T6 aluminium. The door was sealed via a dovetail groove and O-ring, which included a rectangular viewport with safety cover. All these properties allowed us to reach ultra-high vacuum levels.

### 2.2. Commercially Supplied: Manipulator

As mentioned above, the GLAD technique was based on the control of three parameters: rotation speed-φ, angle-α, and temperature. The system was based on the ECR (ECR-UHV-20532-001) manipulator (Figure 4b). To enable a high-precision deposition with repeatable conditions, the parameters were controlled by a controller, including both hardware and software elements; this is discussed below in detail.

The first element was responsible for rotation. It consisted of a brushless DC motor with a STM32 NUCLEO-H745ZI-Q (STMicroelectronics, Geneva, Switzerland) as the controller. The BLDC58-35LEB Series engine (Allied Motion Technologies, Amherst, NY, USA) was 24 (V) DC powered, and had integrated drive electronics that could be easily controlled with two parameters. It featured up to 50 watts of continuous output power, variable speed proportional to the control signal, and different directions of rotation. In accordance with the use of brushless technology, it was characterised by a long working life and small failure rate. The maximum speed was 3650 rpm and the minimum regulated speed was 1000 rpm. This speed range was too high for the GLAD process, so there was a reduction gear added, which allowed us to reduce velocity at a ratio of 50:1.

To enable keeping a constant rpm (φ), a BLDC58 DC motor was used. This engine was controlled by two parameters. The first was the digital signal LOW/HIGH to set the direction of rotation. To set the required speed, the engine was controlled by voltage in the range of 0 to 5 (V). To control the speed, the engine had an integrated encoder. Using the STM32 Nucleo microcontroller, which had built-in timers, we could count the real rpm from the output rectangular signal. With the digital-to-analogue converter (DAC) module, which was communicated by I2C, we could precisely set the required voltage. The DAC module resolution was set to 12 bit, which enabled a high level of accuracy.

**Figure 5:**
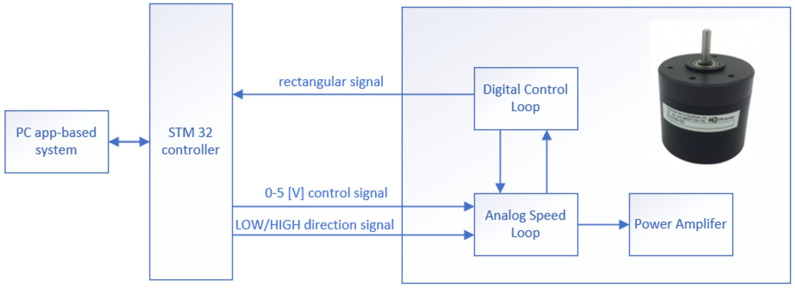
Rotation speed controller connection.

The connection diagram of these elements is presented in Figure 5.

The motor was controlled by STM32 NUCLEO-H745ZI-Q. This microcontroller was a series of boards with STM32 microcontrollers in LQFP144 packages. The boards were equipped with an ST Zio connector, which was a development of the connector known from Arduino boards. Nucleo-144 boards were equipped with a built-in STLINK-V3 programmer/debugger; libraries and examples were available for them. The STM32 Nucleo-144 provides users with an affordable and flexible way to test new concepts and build prototypes with a choice of various combinations of performance and power consumption features provided by the STM32 microcontroller. It contains two cores: ARM Cortex M7 32-bit with clock frequency 480 (MHz) and ARM Cortex M4 240 MHz, 2 (MB) flash program memory, 1 (MB) RAM, 16/32-bit timers and quartz resonator 32,768 (MHz).

**Figure 6:**
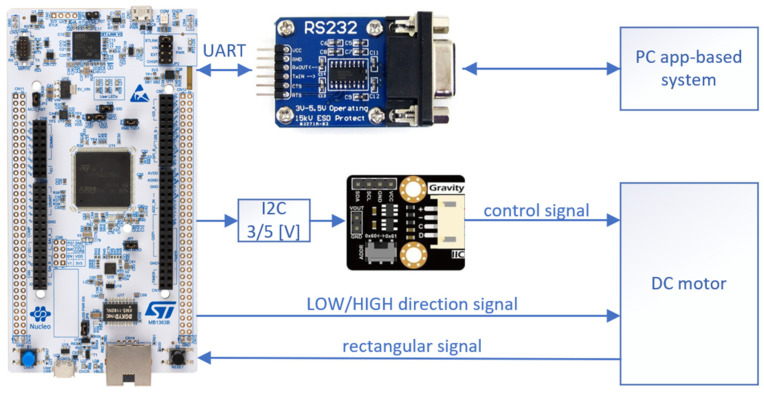
Scheme of connections between microcontroller and peripheral modules.

Using built-in timers, the module could convert the rectangular signal from the BLDC58 into frequency and, further, it was processed to calculate the RPM of the motor. Unfortunately, the digital-to-analogue converter of the microcontroller was of no use in the control of rpm because the maximum voltage it could generate was 3.3 (V). This was not enough for the engine and we needed an additional DAC module where we could obtain values up to 5 (V). The solution was MCP4725 and a logic level converter of 3.3/5 (V). The last module that extended microcontroller capacities was the RS232 to UART converter with the SP3232 controller. The connection scheme is shown in Figure 6. The module was developed by our team.

The second part of the manipulator was angle α controller. It contained two elements, a Sim-Step controller and a stepper motor, both from McLennan Servo Supplies (Aldershot, UK).

Sim-Step is a system solution for programmable positioning. It has an integrated high-efficiency Bi-polar drive stage, a 400 step/rev. motor resolution which provides increased smoothness, an internally adjustable phase current from 0.5 to 3.5 amps, and an integrated motion controller.

**Figure 7:**
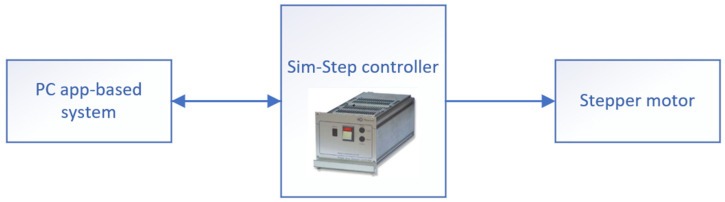
Angle controller connection.

In order to precisely set up the required angle α, a stepper motor was used. This is a brushless DC electric motor that divides a full rotation into a number of equal steps. The motor’s position could be commanded to move and hold at one of these steps without any position sensor for feedback, as long as the motor was correctly sized to the application with respect to torque and speed. The stator had ten magnetic poles with small teeth, each pole being provided with a winding. Each winding was connected to the winding of the opposite pole so that both poles were magnetised in the same polarity when a current was sent through the pair of windings, as shown in Figure 7. A Nanotec ST5918 device was mounted in the manipulator, which was characterised by hybrid construction featuring high-energy magnets, 400 step/rev resolution using a packaged drive, and optimised for high-torque output.

In this configuration (Figure 7), the controlled application developed by our team was directly connected to the stepper motor adjuster. The accuracy of the positioning was very important to obtain a proper structure. Using this type of engine allowed the angle to be precisely set.

**Figure 8:**
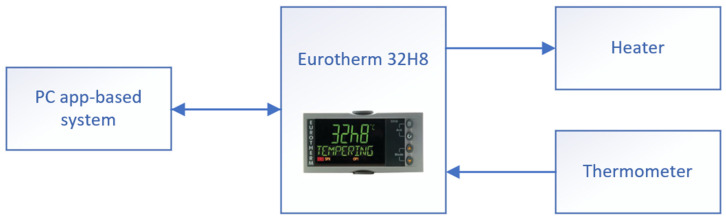
Temperature controller connection.

The third important parameter was the temperature of the substrate; herein, the substrates could be heated up to 850 °C. The temperature needed to keep stable during the deposition process. In this case, a commercial Eurotherm 32H8 device was used (Eurotherm, Worthing, UK). The parameter could be configured directly from the application developed by our team, as shown in Figure 8.

### 2.3. Commercially Supplied: Magnetrons

In this study, TRS2 magnetron sputtering sources from the Kurt J. Lesker Company were used. The magnetrons were indirect-cooled with an integral anode shielding; such design is optimal for carrying out scientific and industrial research. The magnetrons were equipped with sputter cathodes (2 inches in diameter) that are functional with virtually any material, and have a wide pressure range operating up to 1.33⋅10^−8^ (mbar), and have low outgassing.

### 2.4. Commercially Supplied: Vacuum Pump

There were two vacuum pumps: a YTP1100-4F16A rotary pump (Eurovacuum B.V., Reeuwijk, The Netherlands) and a TURBOVAC 360C turbomolecular pump (Leybold GmbH, Cologne, Germany). The TURBOVAC is a pump featuring grease-lubricated bearings.

**Figure 9:**
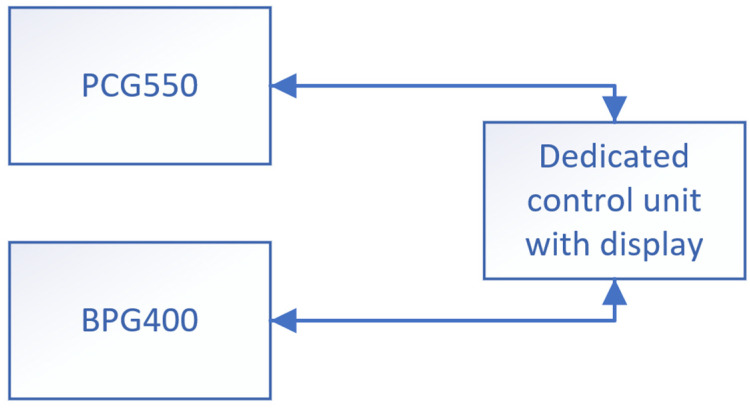
Vacuum probe controller connection.

A set of two vacuum probes was used to control the pressures before and after the turbomolecular pump (Figure 9); these were a Pirani Capacitance Diaphragm Gauge PCG550 and a Bayard–Alpert Pirani Gauge BPG400, both from INFICON (Bad Ragaz, Switzerland). The first had two sensors: a piranha sensor and a membrane capacitive sensor. The measuring range was from 5 × 10^−5^ to 1500 (mbar). The second used the Bayard–Alpert measurement system and the Pirani measurement system. It had a much more accurate measuring range from 5 × 10^−5^ to 1500 (mbar).

### 2.5. Developed by Our Team: App-Based System

**Figure 10:**
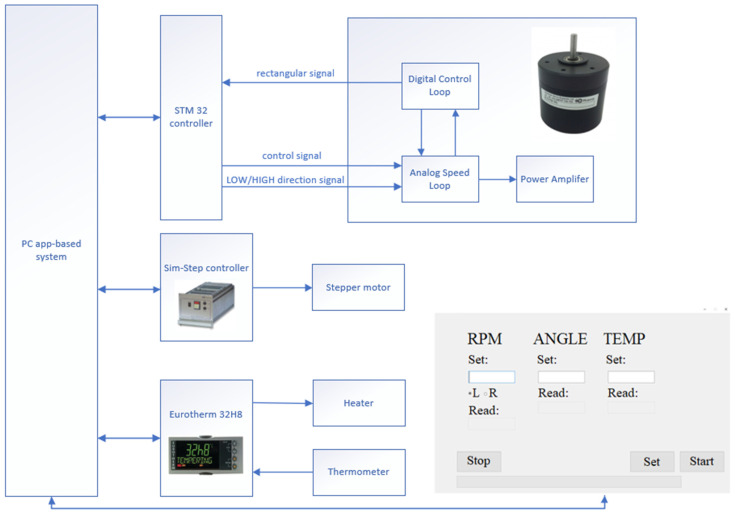
The connection of the GLAD machine controller.

This dedicated application, which was developed by our team, was written in the Python programming language. It was configured to serve as a GUI (graphical user interface) for the operator. In the application, parameters such as rotation speed φ rpm, rotation direction L/R (which refers to: L—left; R—right), angle α and the temperature of the substrate could be set. The application also provided information about the actual measured rpm, angle, and temperature. The connection of all components to the application is shown in Figure 10.

## 3. Experimental Details

The metal-oxide-based gas-sensing materials were obtained in the above-mentioned magnetron sputtering system which was equipped with three magnetron sources that enabled deposition in a single or parallel mode when multilayer oxides were required. The system was pumped down to 5 × 10^−6^ mbar for base pressure and the deposition was conducted at 2 × 10^−2^ mbar, which was previously confirmed. During these experiments, copper and tungsten targets (both from Kurt J. Lesker Company) of 50 mm diameter (5N purity) were employed for reactive sputtering at 80%Ar/20%O_2_ gas mixture. The flows of argon and oxygen were controlled by mass flow controllers (1179B from MKS Instruments, Andover, MA, USA). The substrate tilt angle varied from 75° to 85°, and the sample rotation at a speed of 0, 10, and 20 RPM was stabilized with the utilization of the developed algorithm. The deposition power was kept constant at 50 W, and deposition time varied depending on the material, i.e., 40 min and 90 min for CuO and WO_3_, respectively. The distance between magnetron targets and substrate holder was constant and it was 200 mm. The layers were deposited onto silicon substrates (Si-Mat, Kaufering, Germany), quart JGS-2 (Continental Trade, Warsaw, Poland), and DRP sensors with electrodes (Metrohm, Oviedo, Spain). As was already mentioned, the deposition temperature is one of the crucial parameters when nanostructures such as nanocolumns and nanopillars are planned. In our works, we based our work on the model proposed by Movchan and Demchishin, which was based on the morphological characterization of several vacuum-deposited materials [40]. Within this model, three zones I, II, and III (structure zone models) related the film microstructure to the homologous temperature T_s_/T_m_, where T_s_ is the substrate temperature and T_m_ is the melting point of the deposited material. Most relevant to GLAD was the low-temperature zone I regime, corresponding to T_s_/T_m_ < 0.3, and more can be found in reference [40].

## 4. Results and Discussions

The stability of the process parameters directly affected the parameters of the sputtered layers. Unstable conditions during the GLAD process could result in an uneven build-up of material as the tallest columns grew at the expense of the smaller elements [41]. Previously, we tested the impact of various argon–oxygen mixtures on copper/copper oxide growth [42], the impact of GLAD sputtering parameters on the crystal structure, morphology, and gas-sensing properties of the tungsten trioxide films [12], the influence of the GLAD nuclei process to ultra-thin copper oxide films [3], as well as to the gas-sensing properties of the copper oxide–tin oxide heterostructures [43]. Herein, a brief report on the deposition system and further improvements was proposed and the given sensing results are shown to confirm the previously obtained results.

**Figure 11:**
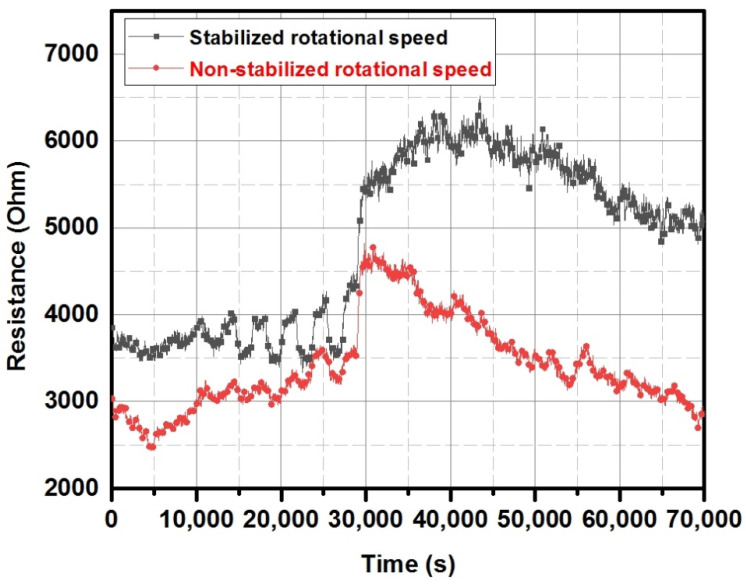
The sensor layers obtained in stabilised rpm conditions (black trace) and not-stabilised (red trace) exposed to variable NO_2_ concentrations and atmospheric air.

In the presented system, a controller for the brushless motor was used to control the rotation of the substrate table. The engine was coupled with a manipulator through a gearbox, reducing the speed by a 50:1 ratio. This induced the engine to work in a relatively high-speed range. An optical encoder generating 36 pulses per revolution was a source of information about the rotor speed. The signal, thus, obtained was not stable and may have caused some fluctuations during the sputtering process. In order to eliminate this effect, a controller was used that acquired information from both the encoder connected to the STM32 microcontroller timer and an oscilloscope. The feedback obtained in this way corrected the control signal; thus, reducing the instability of rotation. Figure 11 shows the results of tests of layers obtained in a similar process, with and without the use of additional stabilisation. The sensor layers were placed in the test chamber, where gas (nitrogen dioxide) was dosed in a variable concentration, as previously presented [43].

As can be observed, the layers exhibited a similar electrical resistance. After the gas-dosing (30,000 s), the sensors were exposed to the atmospheric air to simulate real working conditions. In the case of a sensor deposited at the constant rotation value, the signal was characterised by a much more stable reading of the baseline. Furthermore, the reading of the resistance change as a function of the variable gas concentration on the stabilised sample was much higher, and, therefore, exhibited a higher stability in the practical application.

**Figure 12:**
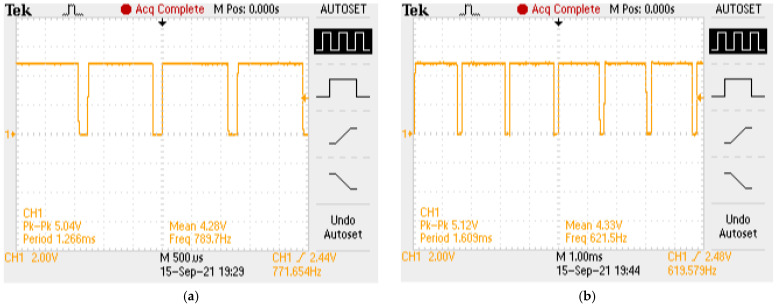

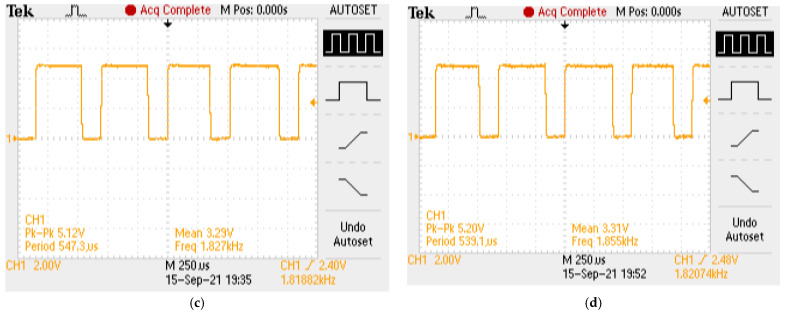
Example results of experiments: (**a**) 20 (rpm), clockwise revolutions; (**b**) 20 (rpm), anticlockwise revolutions; (**c**) 60 (rpm), clockwise revolutions; (**d**) 60 (rpm), anticlockwise revolutions.

As mentioned above, the controlling of the deposition parameters was a crucial part of the developed system. Thus, reference measurements were conducted to analyse the readings delivered by the STM32 microcontroller. In this case, the oscilloscope was set to measure the frequency of the rectangular signal output. The read-out from both devices was recorded. The rotation speed was set in multiples of five, and the rotation direction was set in both clockwise and anticlockwise revolutions. Example results from the oscilloscope are presented in Figure 12, and example data from both devices are given in Table 2.

**Figure 13:**
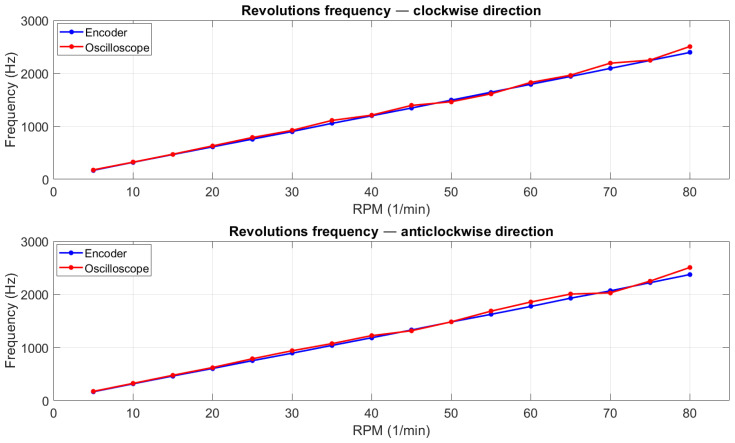
Plots with curves representing encoder and oscilloscope measurement values of rotation frequency.

Figure 13 presents a graph of the results, split by rotation direction.

**Figure 14:**
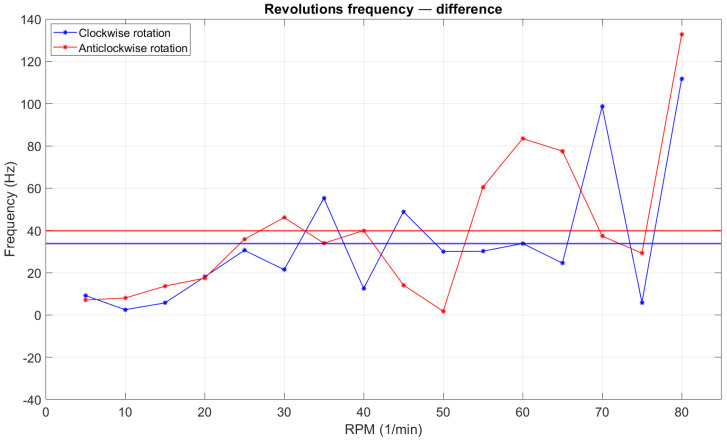
Plots with polyline of absolute value of the difference between encoder and oscilloscope values; straight line shows value of absolute error.

According to the data presented in Figure 14 and Table 3, it could be conducted that the utilisation of the STM32 microcontroller as a major element of the device enabled measurements with high precision. The error level was approximately 3% and this was acceptable, taking into account the measurement environments, such as high vibration. Additionally, the engine did not spin evenly at each stage of rotation. This effect was observed during the experiment. In Figure 12, the oscilloscope provides information about the frequency of the plot in the single mode (the value shown in the chart area) and instantaneous frequency (the value shown in the bottom right corner of oscilloscope screen). The differences in frequency values for the same revolutions could be up to 100 (Hz). However, our results confirmed that the rotation speed was calculated correctly with the utilisation of microcontrollers. To set the revolutions, the relevant formula was derived on the basis of the engine documentation and theoretical calculations. The next experiment was designed to investigate the dependency of set and actual rotation speeds. To make it accurate, 10 values of actual rpm in the range of 5 to 80 were recorded within a one-second period. The engine was stopped between each rpm value.

**Figure 15:**
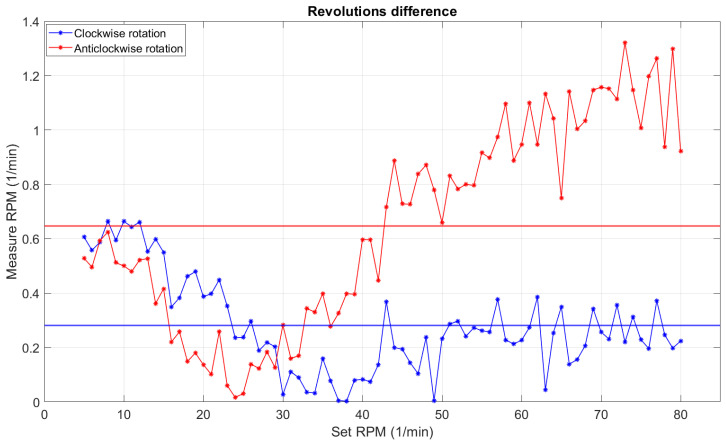
Plot of the values of differences between set and actual rpm; the straight line shows absolute error.

Figure 15 shows that the actual rotation could not be used as for setting the rotation value (without calibration) and after 50 rpm, a significant leap could be seen. The anticlockwise rotation was subject to a greater error between the assumed and actual value than the clockwise rotation. Using the actual rotation speed data obtained from the test, the coefficients of the fourth-degree polynomial, which was the correction function for the previously calculated formula, were calculated with the help of the approximation function separately for both directions. The new formula was tested in the experiment.

**Figure 16:**
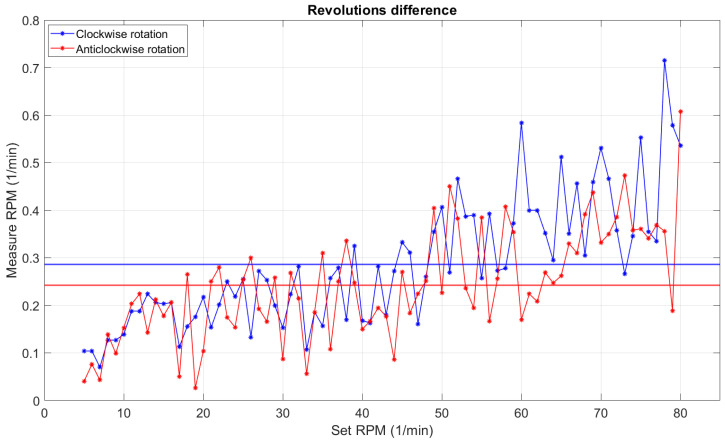
Plot of the values of differences between set and actual rpm; the straight line shows absolute error.

The results presented in Figure 16 showed that the correction function had served its purpose. The difference chart was flattened and values were more concentrated on one level. To check the impact of the algorithm, the absolute and relative errors were calculated and compared in Table 4.

The calculated errors showed a significant improvement in the stability and repeatability of the rotation speed. It could be observed that the absolute error in the clockwise direction had not changed, opposite to the anticlockwise rotation, where we could observe a significant decrease. The relative error for both directions was far superior after correction. It can be seen from Figure 16 that the points were not scattered. The most important problem in this experiment was the non-repeatability of the tests due to the inaccuracy of the engine. However, the average error did not exceed 0.5 rpm, and the maximum error values in this test did not exceed 1 rpm, which was a satisfactory result.

**Figure 17:**
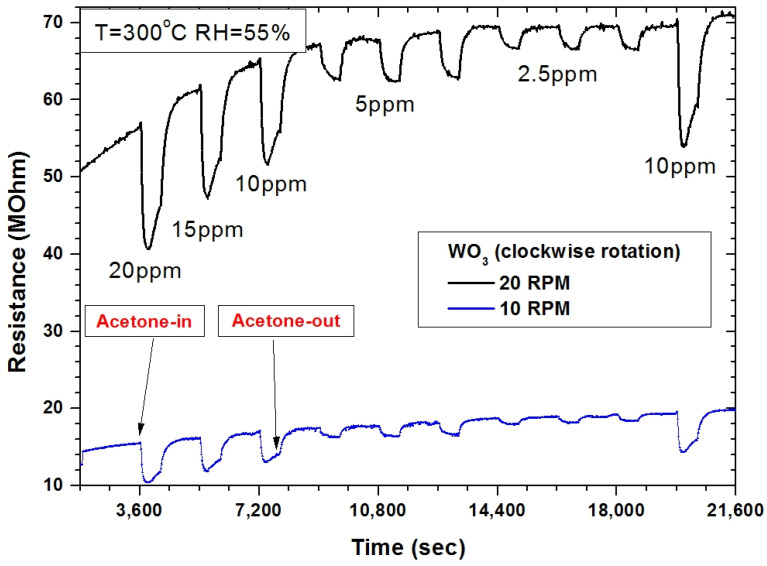
The gas-sensing characteristics of WO_3_-based gas sensor deposited with various rotations with stabilization, under exposure to acetone in the range of 2.5–20 ppm, at 300 °C and 55% RH.

In Figure 11, an example of the effect on the rotation stabilization for resistance changes of the CuO-based sensor for outdoor nitrogen dioxide changes is presented. Although, the gas-sensing applications required a stable sensor response in various conditions, for example, for volatile organic compound (VOCs) detection. Such applications attract more attention nowadays due to the possible application in medical diagnosis. Recently, a number of papers was published, where VOC detection was suggested as a supplementary tool for medical diagnosis and screening, including acetone detection in the exhaled human breath [44,45,46,47,48,49,50,51,52,53]. It is worth mentioning that medical applications require high standards for sensor quality, and in terms of screening tests, a number of devices with repeatable responses would be required. Therefore, the stabilization of deposition parameters was more than crucial. Figure 17 presents the gas-sensing characteristics of the WO_3_-based gas sensor deposited in GLAD deposition (a deposition angle of 80° was chosen based on the previous experiments [54]), with 10 and 20 rpm with stabilization. The sensor was tested under exposure to acetone at 2.5 ppm, 5 ppm, 10 ppm, 15 ppm, and 20 ppm. The measurement conditions were kept constant during the measurements, i.e., 300 °C and 55% relative humidity (RH). Interestingly, the sensor deposited at a lower rotation (10 rpm) exhibited a lower base resistance and further lower responses in comparison to the sensor deposited at the maximum rotation (20 rpm). It can be explained by the effects resulting from the GLAD deposition, where shadowing effects occurred. Explained previously in [5], the optimal rotation could be estimated taking into account the base material (in this case, tungsten deposited in the reactive mode), dimensions of the chamber, deposition temperature, and power. Furthermore, the sensors deposited without rotation exhibited no response to acetone in the above-mentioned range; the range covered acetone concentrations in the exhaled human breath [54].

**Figure 18:**
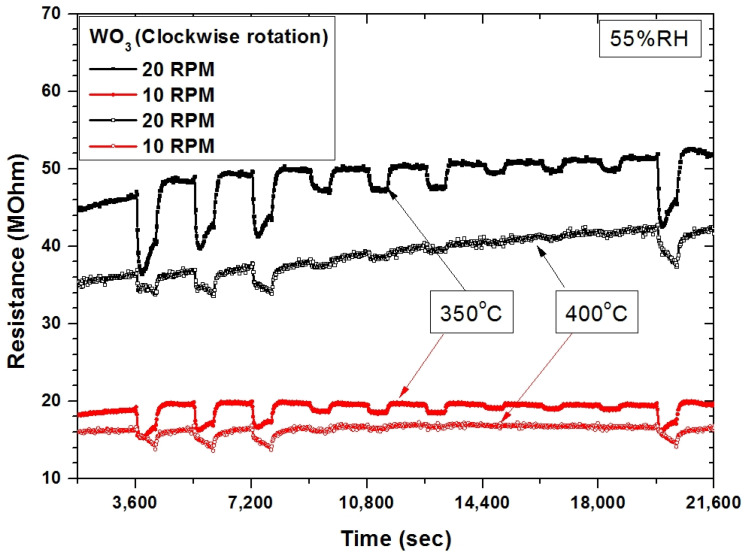
The gas-sensing characteristics of WO_3_-based gas sensor deposited with various rotations with stabilization, under exposure to acetone in the range of 2.5–20 ppm, at 350 °C/400 °C and 55% RH.

In 2016, Rydosz et al. [55] showed that WO_3_-based gas sensors deposited with the utilization of the GLAD technique exhibited the highest responses when deposited and measured at 300 °C, and an additional increasing or decreasing deposition temperature did not improve the gas-sensing responses. A similar effect was observed during this study, where an increasing measurement temperature resulted in decreased responses. Interestingly, below 300 °C, the sensors did not respond to acetone. The differences between these studies rely on the deposition equipment, as in [55] a home-made system was used, while, herein, we used a commercially available system with additional stabilization. The obtained results confirmed that the optimal deposition temperature for the WO_3_-based gas sensors (deposited in the form of nanostructures) to acetone was around 300 °C, while the optimal measurement temperature depended on the gas sensor substrates, and for WO_3_-based sensors, it usually was in the 300–400 °C range. Figure 18 shows the gas-sensing characteristics of the WO_3_-based gas sensors deposited with various rotations with stabilization under exposure to acetone in the range of 2.5–20 ppm, at 350 °C/400 °C and 55% RH. The responses were higher when sensors were heated up to 350 °C. Additionally, the measurements were conducted in the same gas-sensing setup; therefore, there was no bias from the various systems/methods used during the measurements. It has to be underlined that to open the commercialization road from research outcomes to practical application, the technology should be scalable and repeatable. If not, obtained results will never pass the obstacle called the valley of death.

## 5. Conclusions

Developments in nanotechnology have paved the way for the industrial revolution in many fields, although in electronics, the progress has been obvious. The crucial element of nanotechnology is the deposition of the thin and ultra-thin films of metals, metal oxides, and metal nitrides. The controlling of the deposition has to be performed with high precision in order to obtain a high-quality film. The problem increases when nanostructured films need to be deposited. As a solution, the glancing angle deposition technique was developed. GLAD offers the possibility to fabricate nanostructured columnar thin films without using any templates and which can be applied in various deposition systems. GLAD requires a minimal set of parameters with regard to the tilt, rotation, and substrate temperature. In this study, we showed an easy and cheap realisation of a microcontroller-based system that enabled the high-precision control of the GLAD parameters. The obtained results confirmed that films deposited with the utilisation of such a controller exhibited a higher stability. Further tests are planned with various conditions to obtain films with more sophisticated structures that would not be possible to obtain without high-precision control.

## Figures and Tables

**Table 1:** Description of the elements listed.

Letter	Description
(a)	Chamber
(b)	Manipulator
(c)	Magnetrons
(d)	Vacuum pump
M1	DC motor
M2	Stepper motor

**Table 2:** Comparison of frequency value.

Rotation Speed (rpm)	Encoder Frequency (HZ), Clockwise Revolutions	Oscilloscope Frequency (HZ), Clockwise Revolutions	Encoder Frequency (HZ), Anticlockwise Revolutions	Oscilloscope Frequency (HZ), Anticlockwise Revolutions
20	611.61	629.60	604.11	621.50
60	1793.19	1827.00	1771.59	1855.00

**Table 3:** Values of absolute and relative error; oscilloscope was used as a reference.

	Absolute Error	Relative Error
Clockwise directions	33.66 Hz	2.62%
Anticlockwise directions	39.89 Hz	3.11%

**Table 4:** Absolute and relative errors of the differences between set and actual rpm before and after correction.

	Absolute Error		Relative Error	
	Before	After	Before	After
Clockwise directions	0.2813 RPM	0.2862 rpm	1.5%	0.80%
Anticlockwise directions	0.6475 RPM	0.2419 rpm	1.93%	0.69%

## Data Availability

The data presented in this study are available on request from the corresponding author.
